# Harnessing engineered virophages: a novel frontier against antibiotic-resistant superbugs

**DOI:** 10.1097/MS9.0000000000004322

**Published:** 2025-11-20

**Authors:** Muhammad Talha, Maliha Khalid, Hamza Muhammad Jafar, Aminath Waafira

**Affiliations:** a King Edward Medical University, Lahore, Pakistan; b Jinnah Sindh Medical University, Karachi, Pakistan; c The Maldives National University, Malé, Maldives

**Keywords:** antimicrobial resistance, engineered virophages, multidrug-resistant bacteria

*To the Editor,*

Antibiotic resistance is perhaps the biggest challenge facing contemporary medicine. Multidrug-resistant (MDR) bacteria probably brought 1.27 million deaths worldwide in 2019 alone, and that number is undoubtedly going to increase rapidly in the absence of newer modalities^[1]^. The traditional antibiotics will soon become less effective against superbugs such as carbapenem-resistant Enterobacteriaceae and *Pseudomonas aeruginosa*. Thus, there is a need to devise new medicines. Novel engineered virophage therapies have been recently developed to neutralize resistant bacteria. These therapies use genetically modified bacteriophage agents and have been earmarked as viable solutions with profound prospects in winning the battle against antimicrobial resistance (AMR). Distinct from virophages, which attack other viruses, engineered bacteriophage therapy has been developed against those resistant bacteria. Genetically modified bacteriophage viruses infect specific bacteria; use of these viruses is emerging as a possible solution with high potential to address AMR^[1]^.

Bacteriophages (phages) are viruses that target bacteria with high specificity while leaving human cells and commensal microbiota unimpaired. Advances in synthetic biology have allowed for engineered phages to bypass bacterial defense systems, such as Clustered Regularly Interspaced Short Palindromic Repeats–CRISPR-associated (proteins), to deliver lethal genetic payloads or disrupt the genes that confer resistance^[2]^. For clarity, the term “engineered bacteriophage therapies” represents these modified phages, distinguishing them from virophages, which are not the focus of this work. A recent study indicated that modified virophages can hit MDR strains of *P. aeruginosa*, commandeering their replication machinery to lyse bacteria while concurrently degrading antibiotic resistance genes and thus effectively reversing their resistance phenotypes^[3]^. This dual-action mechanism sets virophage therapy apart from traditional antibiotics and early attempts at phage therapy that suffered limitations imposed by bacterial immunity and narrow host ranges.

**HIGHLIGHTS**

- Genetically engineered virophages offer a dual-action strategy to both lyse multidrug-resistant bacteria and degrade their resistance genes.
- Preclinical studies show improved survival and reduced bacterial loads using virophage cocktails in multidrug-resistant infections.
- Virophage therapies preserve host microbiota and offer a slower resistance evolution profile compared to traditional antibiotics.

The therapeutic potential of virophages is supported by a lot of experimental models. In murine infection studies, a cocktail of phages targeting Klebsiella pneumoniae and Acinetobacter baumannii achieved a considerable decline in bacterial load and increased survival rates when compared with untreated controls^[4]^. With this continued co-evolution with bacterial populations, phages might take resistance development at a slower pace compared to antibiotics, allowing for flexible and sustainable treatment schedules. In addition, the specificity of phage therapy protects the host microbiome, which, in turn, minimizes the risk of secondary infections and dysbiosis attributed to broad-spectrum antibiotics. But long-term effects on the microbiome and the possibilities of evolving resistance will need further research because most of the evidence so far has been from short-term studies.

Despite these advances, several challenges remain to be overcome before virophage therapies could find general application. The complexity of phage-host interactions requires extensive characterization for efficacy and safety to be assured within various groups of patients. Large-scale, randomized clinical trials are highly needed to ascertain standardized dosing, delivery methods, and long-term safety profiles because much of the currently available evidence is preclinical^[5]^. Regulatory pathways through which phage therapeutics would be approved are still being established; thus, large, randomized clinical trials are urgently needed to give direction for standardization in dosing and delivery methods and long-term outcomes^[5]^. In light of the escalating AMR threat, however, funding agencies, policy makers, and clinicians must establish and integrate virophage research into antimicrobial stewardship programs. Our work is in line with the TITAN Guidelines on the need for transparency in artificial intelligence use in healthcare^[6]^.

To conclude, engineered bacteriophage therapies present an effective opportunity in fighting antibiotic-resistant superbugs. Their specificity, two modes of attack, and potential for a delayed-onset resistance development could favor them over conventional antibiotics. And yet, clinical acceptance will depend on overcoming existing limitations through well-controlled clinical trials, standardized production, and clear regulatory pathways. The medical research community is encouraged to work towards multidisciplinary synergy to overcome these hindrances so that bacteriophage therapy can be integrated into clinical practice and help lessen the burdens of AMR.

## Data Availability

No data were generated for this manuscript.
